# Genetic dissection of protein content in cowpea using custom-made NIRS equations and GWAS as a model for nutritional breeding and undergraduate research training

**DOI:** 10.1093/g3journal/jkag088

**Published:** 2026-04-06

**Authors:** Habib Akinmade, Shiri Ben-Israel, Audrey Ryan, Chase Johnson, Annmary Tharayil, Rebecca Olivia Arias, Rebecca Caroline Ulbricht Ferreira, Claudio Fernandes, Ziynet Boz, Esteban Rios

**Affiliations:** Plant Breeding Graduate Program, University of Florida, Gainesville, FL 32611, United States; Department of Geography, College of Liberal Arts and Sciences, University of Florida, Gainesville, FL 32611, United States; Department of Sustainable Biomaterials and Packaging, College of Forestry, Wildlife and Environment, Auburn University, Auburn, AL 36849, United States; Commercial Operations Department, Wish Farms, Plant City, FL 33563, United States; Department of Nutrition and Food Sciences, Texas Woman's University, Denton, TX 76204, United States; Plant Breeding and Plant Genetics Graduate Program, University of Wisconsin-Madison, Madison, WI 53706, United States; Molecular Biology and Genetic Engineering Center (CBMEG), University of Campinas (UNICAMP), Campinas, Sao Paulo 13083-875, Brazil; Analytics Department, GDM Seeds, Campinas, Sao Paulo 13091-611, Brazil; Department of Agricultural and Biological Engineering, Institute of Food and Agricultural Sciences, University of Florida, Gainesville, FL 32611, United States; Plant Breeding Graduate Program, University of Florida, Gainesville, FL 32611, United States; Agronomy Department, University of Florida, Gainesville, FL 32611, United States

**Keywords:** crude protein, GWAS, NIRS, *Vigna unguiculata*

## Abstract

As the demand for plant-based nutrition increases, improving the protein profile of legumes like cowpea has become a breeding priority. Cowpea, a multiuse legume and staple in many low-income regions, provides important dietary protein that can help meet the demand in our growing population. Our research used genome-wide association studies (GWAS) and phenomic tools to investigate the genetic architecture of seed protein content in cowpea and integrated 4 cohorts of undergraduate researchers through a USDA-AFRI REEU program. Using wet chemistry and near-infrared spectroscopy (NIRS), we assessed crude protein (CP) within the University of California Riverside Minicore collection, developed and validated a custoMED-made NIRS calibration equation for CP (*R*^2^ = 0.86), and performed GWAS with ∼41k single-nucleotide polymorphisms (SNPs). Significant SNPs associated with protein content were identified on chromosomes 1, 3, 7, 10, and 11, and candidate genes were linked to functions including nutrient transport, stress response, and seed storage protein regulation. These results provide a foundation for future marker validation and functional studies, and demonstrate the value of pairing trait discovery with undergraduate training.

## Introduction

With the world population set to reach 9.6 billion in 2050 (United Nations 2017), improving the nutritional quality and climate adaptability of food crops is important to meet the increasing pressures on global food systems. Legumes are critical in achieving this, given their diverse roles that contribute to food security and agricultural sustainability (Foyer et al. 2016; Stagnari et al. 2017). Not only are legumes a good source of dietary protein, fiber and other essential micronutrients, but they also contribute to soil health, improve biodiversity in cropping systems and support low-input, environmentally sustainable farming (Vance et al. 2000; Garrigues et al. 2012; Nedumaran et al. 2015; Reckling et al. 2014; Praharaj and Maitra 2020). Even with these benefits, nutritional and yield gains in many legume crops have trailed those of major cereal crops, mostly due to historical underinvestment in breeding and trait discovery, and dominance of cereals on arable land (Graham and Vance 2003; Siddiq et al. 2012; Varshney et al. 2013; Navin Venketeish et al. 2024). Addressing these issues will require increased investment and adoption of new breeding approaches that are tailored to improving legumes.

Cowpea (*Vigna unguiculata* [L.] Walp.) is a multiuse legume crop of global importance due to its nutritional value and stress resilience. It is an important source of plant-based protein, fiber, essential minerals like magnesium and iron, which makes it a go-to crop in low-income regions (Boukar et al. 2019; Ajmera 2024). In addition to human nutrition, the crop also benefits agroecosystem through biological nitrogen fixation, which reduces the reliance on fertilizers and lowers investment costs for farming systems (Poudel et al. 2025). Its adaptability to nutrient-deficient soils, heat and drought tolerance also makes it very useful in semiarid and tropical environments, where it continues to aid food and nutritional security (Hall 2012; Adair 2023; Raizada et al. 2023; Shevkani et al. 2025). Cowpea's quick growth and ground covering attributes also make it a good cover crop, helping to conserve soil moisture and controlling weeds (Harrison et al. 2006; Herniter et al. 2020; Dareus et al. 2021; NRCS 2024). Cowpea is also compatible in cereal intercropping systems, which gives room for diversified agricultural practice, increasing land productivity (Ongom et al. 2023; Sanfo et al. 2023). Due to these functions, cowpea remains a strong candidate to fit into strategies that can help cushion the effects of the climate change intensification.

Protein content is a key trait of interest due to its direct relevance to diets. In different parts of sub-Saharan Africa, Latin America and south Asia where cowpea is consumed daily, it is one of the most affordable sources of protein (Jayathilake et al. 2018). The protein content of cowpea ranges from 20% to 30% (Carvalho et al. 2012; Weng et al. 2017; Dakora and Belane 2019; Weng et al. 2019), helping to resolve major gaps in protein consumption, especially in starchy diets (Mekonnen et al. 2022), and among vulnerable communities with limited access to animal-based protein (Ajmera 2024; Shevkani et al. 2025). This nutritional function is particularly important for children, women and other marginalized populations that suffer from protein-energy deficiencies (Trehan et al. 2015; Langyan et al. 2022; Lindsay 2024). Cowpea also offers a good alternative for soybean (*Glycine max* L.) in meals, providing similar protein levels for people with soy allergies (Boukar et al. 2019). In addition to its value for human foods, protein content is also essential for animal feed and market grading in some industrial systems (Mekonnen et al. 2022). Unlike protein from animal sources, plant-based proteins do not pose the challenges of limited availability, high costs, and potential health hazards (Sabate et al. 2014; Pojić et al. 2018; Munialo et al. 2025). Finally, like other legumes, is a rich in dietary fiber, essential amino acids, and its proteins are linked with reduced risks of heart diseases, obesity and type 2 diabetes (Jayathilake et al. 2018; Guasch-Ferré et al. 2019; Langyan et al. 2022). Hence, improving cowpea protein contributes not only to nutritional development, but also to better public health outcomes and a more socially impactful agriculture.

The chemical composition of food and other agricultural products has been traditionally conducted using classical methods like Kjeldahl, Soxhelt, and Dumas (Wybraniec et al. 2013; Weng et al. 2017; Mæhre et al. 2018; Lopez-Bascon and de Castro 2020; Auria et al. 2023). However, these methods are time-consuming, labor-intensive, use harmful chemicals, all of which pose economic and environmental challenges, especially when applied to large-scale quality assessment of agricultural products (Hayes 2020; Levasseur-Garcia et al. 2024). As the agricultural industry grows faster methods to determine the chemical properties of products are needed. One of such methods is the near-infrared reflectance spectroscopy (NIRS), a much faster, less-destructive, and cost-effective method for estimating protein content in seeds (Agelet et al. 2012; Venkatesan et al. 2020; Kassie et al. 2024). NIRS operates based on both light transmittance and reflectance principles (Agelet et al. 2012) that follow the absorption of near-infrared light by organic molecules, which gives a quick analysis with minimal sample preparation and facilitates the evaluation of large sample numbers (Kays et al. 2000; Ingle et al. 2016; Wold and Løvland 2020; Tonolini et al. 2023). NIRS calibrations have been established to assess grain quality traits in sunflower (*Helianthus annuus*), soybean (*Glycine max*), peanut (*Arachis hypogaea*) (Govindarajan et al. 2009; Agelet et al. 2012; Kassie et al. 2024; Levasseur-Garcia et al. 2024). However, the accuracy of NIRS can be affected by factors like seed coat color, sample heterogeneity, emphasizing the need for precise and crop-specific calibration models for better predictions (Oblath et al. 2016; Weng et al. 2017; Abeshu 2021; Kaur et al. 2024; Levasseur-Garcia et al. 2024). Recent studies have also shown that infrared reflectance spectroscopy models, especially those using partial least squares regression (PLSR), can accurately measure seed protein content in cowpea, with coefficients of determination (*R*^2^) as high as 0.85 showing its usefulness for high-throughput applications in cowpea breeding programs (Muranaka et al. 2015; Ishikawa et al. 2017; Padhi et al. 2022).

Genome-wide association studies (GWAS) have been widely used for the identification of genetic regions that are associated with nutritional traits in legumes, primarily generating candidate loci that inform downstream validation and longer-term breeding efforts for biofortified, protein-rich, value-added varieties. In cowpea, Chen et al. (2023a, 2023b) carried out GWAS and genomic prediction (GP) using 110,155 single-nucleotide polymorphism SNP markers from 161 accessions, identifying SNPs associated with gene *Vigun08g039200*, which encodes proteins involved in nutritional improvement. Weng et al. (2019) assessed protein content in 173 United States Department of Agriculture (USDA) cowpea accessions, and reported a range of 21.8% to 28.9%, showing natural variation in a germplasm as well as identifying high-protein accessions that can be used for breeding. Morris et al. (2020) also evaluated a subset of 111 USDA core germplasm and reported significant correlations between protein content, texture, seed pattern, coat color, and inversely with 100-seed weight, they further suggested that morphological traits can be used as indirect criteria for selection. While GWAS for protein in cowpea is limited, several studies have assessed other mineral components and seed quality traits using high-density SNP panels. Huynh et al. (2024) identified QTL regions associated with sugar-related traits including stachyose content, while Nkurunziza et al. (2025) reported significant marker effects on chromosome 7 associated with biological nitrogen fixation traits such as nodule dry weight. Lo et al. (2018) mapped QTLs linked to pod shattering and pod length and Sodo et al. (2025) detected QTLs controlling pod and peduncle length in nearby regions. Beyond cowpea, GWAS for nutritional quality has been conducted in other legumes. In chickpea (*Cicer arietinum*), Srungarapu et al. (2022) identified 20 marker-trait associations for grain nutrients, with 7 SNPs linked to grain protein. In mung bean (*Vigna radiata*), GWAS detected 43 marker-trait associations related to seed mineral concentrations that include phosphorus, iron, zinc, potassium, and sulfur (Wu et al. 2020), providing potential loci for improving mung bean nutritional quality. In soybean, an important source of plant-based oil and protein, several GWAS experiments have been conducted for nutritive value. Hwang et al. (2014) conducted GWAS on 298 accessions and identified 40 SNPs located in 17 regions in the genome controlling seed protein content, and 25 SNPs located in 13 regions of the genome linked with seed oil content, giving additional knowledge to boost marker-assisted selection for nutritional quality. Zhang et al. (2019) also used both GWAS and linkage mapping with 200 accessions and 308 recombinant inbred lines to identify regions controlling seed protein and oil content in soybean. They reported 25 candidate genes that are putatively controlling proteins and oil metabolism, both located within 2 regions (*qPro15-1 and qPro20-1*). In lentil (*Lens culinaris*), Khazaei et al. in (2017) investigated iron and zinc concentrations with 1150 SNP markers in 138 accessions, discovering 2 SNPs that are significantly associated with iron and one associated with zinc content, traits that are important for biofortification. Recently, Affrifah et al. (2022) carried out a comprehensive review of cowpea's nutritional profile, including processing techniques, protein content, and food/feed potential, further showing the agronomic and economic importance of improving seed protein in cowpea. All these studies contribute to knowledge on genetics of nutritional quality in legumes and points to the utility of GWAS in breeding for nutritive value.

To improve nutritional quality in cowpea, our study aims to evaluate seed crude protein (CP) content in a diverse cowpea germplasm using a combination of wet chemistry and NIRS methods. We also aim develop and assess the predictive ability of custom cowpea-specific calibration model and carry out GWAS to identify SNPs associated with protein content. A unique aspect of this study is the integration of undergraduate training through the USDA-AFRI REEU program, giving hands-on experience in crop phenotyping, spectroscopy, and genetics. Our overall goal is not only to understand the genetic architecture of protein content in cowpea, but to also provide active learning opportunities for training the next generation of scientists in interdisciplinary agricultural research.

## Materials and methods

### Plant material and experimental design

A total of 287 cowpea accessions were used for this study. Of these, 281 were sourced from the University of California Riverside (UCR) Minicore Collection (Muñoz-Amatriaín et al. 2021). The remaining accessions were cultivars commonly grown in the Southeastern USA. A field trial was conducted at the Plant Science Research and Education Unit in Citra, Florida (29.4119°N, 82.1098°W), with a row-column layout and in 2 replications in 2021. Each plot was 3 m in length and 0.6 m in width, with 10 cowpea plants manually sown per plot. Plants spacing was 0.3 m within rows and 0.6 m between rows. In addition to the standard agronomic management, fertilizer application was done at 34 kg per ha (10–10–10; N–P_2_O_5_–K_2_O) at 10 d after sowing for better crop establishment. Weed management was done manually as well as postharvest application of clethodim at 0.56 kg per ha with 5% (v/v) crop oil concentrate at 2 weeks after sowing. Bifenthrin was also applied to reduce insect infestation at 0.45 kg per ha as at when required.

### Protein quantification using wet chemistry

CP was measured in a subset of 103 accessions and 2 replicates in total using a standard nitrogen-based wet chemistry method. Nitrogen content was determined using a modified aluminum block digestion protocol originally described by Gallaher et al. (1975). For each sample, 0.25 g of finely ground tissue was digested in 6 ml of concentrated H_2_SO_4_ and 2 ml of H_2_O_2_, with 1.5 g of a catalyst mixture (9:1 potassium sulfate to copper sulfate). The digestion was performed at 375 °C for a minimum of 4 h. Next, total nitrogen in the samples was quantified using semiautomated colorimetry with a Technicon AutoAnalyzer, according to the method of Hambleton (1977). CP was then calculated by multiplying total nitrogen content by a conversion factor of 6.25. Digestions were conducted at the forage evaluation support laboratory, while nitrogen analysis was completed at the Ruminant Nutrition Laboratory, University of Florida. The wet chemistry data provided reference values for NIRS calibration and comparative analysis.

### Preliminary NIRS evaluation and comparison

An initial subset of 56 accessions were selected for a comparative evaluation of NIRS-based CP content estimation using whole and ground seeds. The NIRS method was carried for each accession out using 100 g of cleaned seed. The intact bulk seeds were initially scanned using the NIR DS2500 (Foss-NIR Systems, Inc., Silver Spring, MD, USA). The same sample was then ground using a coffee grinder and sieved through a 2 mm mesh to ensure that fine particles were used for a second NIRS scan. From this ground material, subsample was collected and used for wet chemistry as described above. Two preinstalled calibration equations from the NIRS Consortium for Forage and Feed (https://www.nirsconsortium.com) were used for the CP estimation, which corresponded to the equations for legume hay (LH) and Corn silage unfermented (CSU). Wet chemistry values were used as the reference standard. Pearson correlation coefficient as well as linear regression were carried out in R version 4.5.1 (R Core Team 2025), using the *stats* package to quantify agreement between each method and wet chemistry.

### Development of cowpea-specific NIRS calibration

To improve the accuracy and biological relevance of CP estimation, we developed a PLSR model with cowpea accessions using both wet chemistry and NIRS spectral data. This model was used to generate cowpea-specific protein estimates for downstream analyses including GWAS, and to evaluate difference relative to the estimates from the LH calibration. Briefly, we first extracted best linear unbiased estimates (BLUEs) for each NIRS wavelength from the replicated spectral data using with ASReml version 4.2 (Butler et al. 2023) package in R, with accession and replication modeled as fixed effects to reduce environmental variation. Corresponding BLUEs for CP content were obtained from replicated wet chemistry data using a similar linear model structure. We then used these paired accession-level datasets to train a PLSR model with the *pls* package in R (Mevik and Wehrens 2007). To optimize the number of latent variables, we used a Monte-Carlo cross validation (Shan 2022), and randomly split the dataset 10 times into 90% training and 10% testing subsets. For each split, we fitted calibration models using 1 to 50 latent components. We accessed the prediction accuracy on the remaining genotypes with Pearson correlation and root mean square error of prediction (RMSEP), which quantifies the average deviation between predicted and reference CP values. To test whether computing BLUEs for both predictors and responses (spectra and wet chemistry) reduced the covariance that PSLR captured, we also tried an alternative approach. From this, we fit the PSLR directly using plot-level spectra data and wet chemistry data, with cross validation blocked by accession. We then computed accession-level BLUEs from the resulting plot-level predictions. An external validation of the cowpea-specific NIRS calibration was done using an independent greenhouse dataset of 5 cowpea accessions grown under 2 environments (hot and cold) with 2 replications per accession. The PLSR model was applied to the spectra to predict CP content and predictions were compared with wet chemistry measurements and estimates from the LH calibration.

### NIRS equation and descriptive statistics

A final set of 287 accessions were estimated for CP content based on preliminary findings. CP content was estimated using both the generic LH calibration and a newly developed cowpea-specific calibration model. Summary statistics (mean, range, standard deviation [SD], and coefficient of variation [CV]) were carried out in R, to assess variation in CP content across accessions and calibration methods.

### Statistical analysis and heritability estimation

All statistical models used in were fitted in R version 4.5.1 (R Core Team 2025). CP values obtained with the LH calibration were used to estimate BLUEs for each accession using a linear model with the following structure:


(1) $$y=1\mu +X\beta +X\gamma +Z_{1}r+Z_{2}c+\varepsilon$$

where y is the vector of the phenotypic vector, *μ* is the overall mean, β is the fixed effects of accession, γ is the fixed effect of replication, r and c are random effects of rows and columns respectively. The design matrices *X*, $Z_{1}\;$ and $Z_{2}\;$ correspond to the fixed and random terms, with $r∼N(0,\sigma_{r}^{2})$ and $c\;∼N(0,\sigma_{c}^{2})$ and $\varepsilon ∼N(0,\sigma_{e}^{2})$. This model was implemented using ASReml-R version 4.2 (Butler et al. 2023), and the same field design and BLUE estimation approach are described in Akinmade et al. (2025). These BLUEs were then used as the input phenotypes for the GWAS.

A second model, treating accession as a random effect, was used to estimate the broad-sense heritability (*H*^2^), calculated as: $H^{2}\;=\frac{\sigma_{g}^{2}}{\left(\sigma_{g}^{2}\;+\;\frac{\sigma_{e}^{2}}{r}\right)}$, where $\sigma_{g}^{2}$ is the genotypic variance, $\sigma_{e}^{2}$ is the error variance, and *r* is the number of replicates. The *H*^2^ estimates represent the repeatability of CP content within the evaluated population and experimental environment based on the phenotyping pipeline used.

### Genotypic data

SNP genotyping of the 287 accessions followed previously described protocols (Muñoz-Amatriaín et al. 2021; Andrade et al. 2023; Akinmade et al. 2025). In brief, the accessions were genotyped using the Illumina Cowpea iSelect consortium Array, which includes ∼51k markers distributed across the cowpea genome. Genotyping was performed at the University of Southern California Molecular Genomics Core Facility (Los Angeles). Raw genotype data were processed with the GenomeStudio software (Illumina Inc.), and SNPs were aligned to the IT97K-499-35 v1.0 reference genome (Lonardi et al. 2019) to assign the physical positions. Markers were filtered by removing loci with more than 25% missing data and minor allele frequency over 5%, consistent with best practices to reduce false positives. After filtering, 41k high quality SNPs were used for further downstream analysis.

### Genome-wide association studies

Two GWAS analyses were carried out to identify the loci associated with CP content in the accessions used. In the first analysis, CP estimates using the commercial LH NIRS calibration served as the phenotype. In the second analysis, CP estimates using our new cowpea-specific NIRS calibration model was used as phenotypes. The analyses were performed using the GAPIT v3 packaged in R (Lipka et al. 2012; Wang and Zhang 2021). We implemented 3 GWAS models: FarmCPU (fixed and random model circulating probability unification; Liu et al. 2016), BLINK (Bayesian-information and linkage-disequilibrium (LD) iteratively nested keyway; Huang et al. 2019), and the multilocus mixed model (MLMM; Segura et al. 2012) to assess the robustness of SNP signals across commonly used multilocus frameworks. FarmCPU and BLINK were the primary models used due to their improved power and control of false positives. MLMM was included as an additional approach, as it accounts for both population structure and kinship through a stepwise regression framework. All models incorporated principal components to control for population structure and a kinship matrix to account for relatedness. We estimated the phenotypic variance explained (PVE) following the equation: *V*_qtl_ = 2freq × (1-freq) × effect^2^, where *V*_qtl_ equals the variance of the QTL effect and freq is the allele frequency. This calculation was based on the variance of the adjusted means for each trait (*V*_pheno_) and follows the approach described by Giordani et al. (2022).

### Identification of candidate genes

To identify putative candidate genes that are responsible for significant SNP associations, we used a narrow genomic window approach, based on previously reported LD decay in the same materials used in this research, where LD decays to *r*^2^ = 0.15 over relatively short distances to Andrade et al. (2023) and Akinmade et al. (2025). Given that the same germplasm and SNP platform were used, this window was chosen to balance resolution and capture of linked functional genes without inflation of candidate regions. Specifically, we searched and identified all annotated genes located within a 20 kb upstream and downstream around each significant SNP based on their physical coordinates in the cowpea reference genome IT97K-499-35 v1.2 (Lonardi et al. 2019). Genome annotation files were obtained from the Phytozome database (https://phytozome-next.jgi.doe.gov/) as described by Goodstein et al. (2012). Functional descriptions and gene ontology (GO) terms that are related to biological processes, molecular functions and cellular components were retrieved for the identified candidate genes within the window using REVIGO tool (Supek et al. 2011). Gene expression patterns were subsequently explored by using the legume information system (LIS) portal (https://mines.legumeinfo.org/vignamine/begin.do), to provide biological context for the identified candidate genes. We focused on the expression profiles linked with protein content and seed development. Genes showing expression patterns related to storage protein accumulation or nitrogen metabolism were considered most relevant for our study.

### REEU undergraduate training framework

This study was conducted within the Circularity and Digitalization Skills REEU Program funded by USDA Agriculture and Food Research Initiative (AFRI)-Education and Workforce Development and hosted at the University of Florida. The program is a 10-week, hands-on, interdisciplinary research experience structured to improve capacity in circular economy principles, digitization tools and improve innovation in agri-food systems. From 2022 to 2025, 4 REEU scholars engaged in this cowpea protein content research project. Students were mentored directly in all key components of the workflow such as seed sample preparation, NIRS data collection, data set organization, and data analysis. Students also received training in the use of R programming language for data analysis, result interpretation, report preparation and presentations. Cohort contributions were in line with key workflow stages. Cohort 1 (2022) contributed to the wet chemistry reference dataset and the preliminary method comparison used to evaluate the whole vs ground seed scans and choose the phenotyping approach for expanded screening. Cohorts 2 and 3 (2023–2024) supported expanded NIRS phenotyping and dataset curation for the full panel used, including standardized seed preparation, spectral acquisition and quality control that produced the accession-level phenotype matrices we used for calibration development and GWAS. Cohort 4 (2025) contributed to downstream statistical analyses (calibration evaluation, descriptive statistics, and GWAS interpretation) and participated in manuscript drafting.

To make sure of data quality and reproducibility, all undergraduate-generated measurements followed standardized protocol and verified through supervisory control. NIRS scans were performed using predefined instrument settings and a subset of samples was rescanned to assess consistency. The data entry and cleaning steps were cross-validated against the raw outputs before they were used in further analysis.

## Results

### Preliminary NIRS evaluation for whole and ground seed

**Fig. 1.**
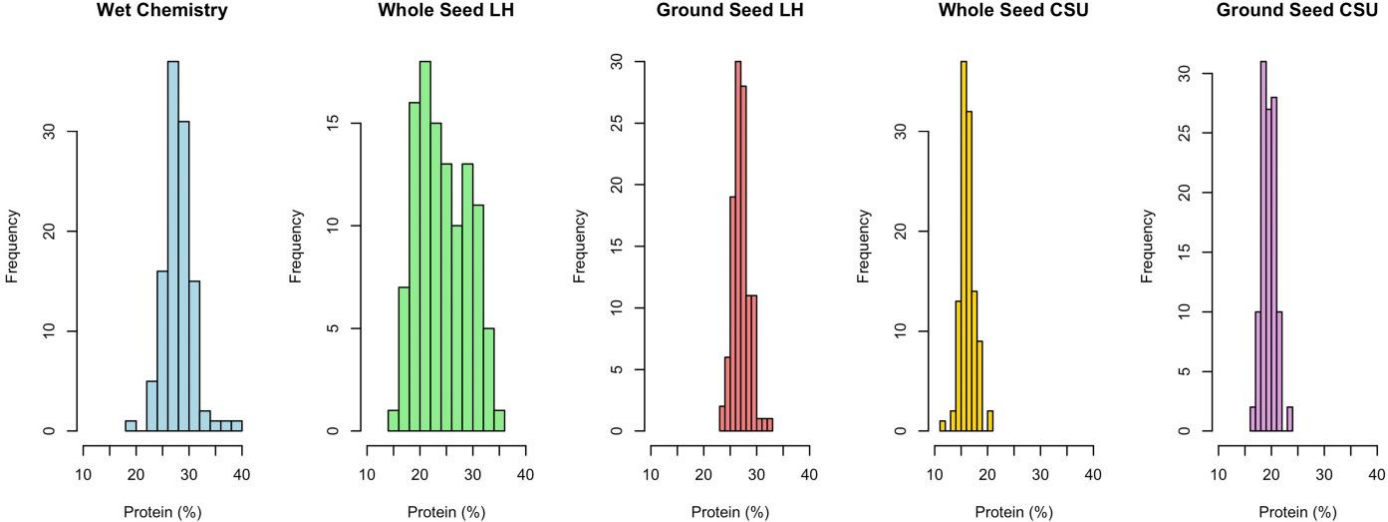
Crude protein content in cowpea seeds estimated by wet chemistry and 4 NIRS-based methods (*n* = 56 accessions). Histograms show protein content (%) obtained from wet chemistry (WC), ground seed using the legume-hay calibration (GS_LH), ground seed using the cowpea-specific CSU calibration (GS_CSU), whole seed using the legume-hay calibration (WS_LH), and whole seed using the CSU calibration (WS_CSU).

CP content measured using wet chemistry for 56 accessions ranged from 21.92% to 37.85%, with a mean of 27.91% (Fig. 1).

**Fig. 2.**
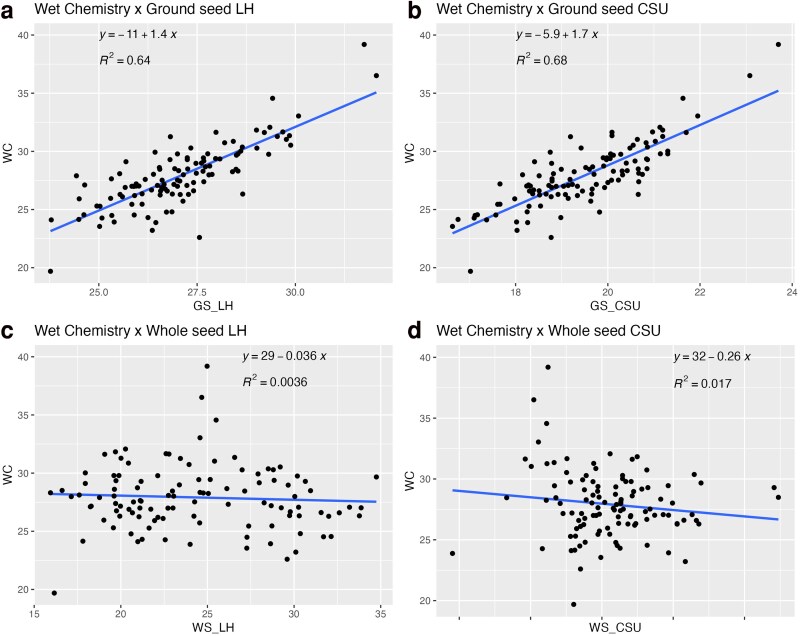
Scatter plots showing the relationship between crude protein content measured by wet chemistry and values predicted using 4 NIRS-based methods. Data points represent individual accessions, and solid lines indicate fitted linear regression models used to assess agreement. Pearson's correlation coefficients (*r*) are shown for each method. Axes represent crude protein content (%) measured by wet chemistry (*x* axis) and predicted by NIRS (*y* axis). Abbreviations: WC, wet chemistry; GS_LH a) ground seed using the Legume Hay calibration; GS_CSU, b) ground seed using the corn silage unfermented calibration; WS_LH, c) whole seed using the Legume Hay calibration; WS_CSU, d) whole seed using the corn silage unfermented calibration.

In comparison, CP predictions from NIRS showed variation depending on seed preparation and calibration equation used. Correlation analysis further supported these observations: ground seeds analyzed with corn silage unfermented (GC_CSU) and GS_LH equations had strong and positive correlations with wet chemistry (*r* = 0.822 and *r* = 0.802 respectively), while both equations using whole seed were not able to estimate CP accurately (WS_LH: *r* = −0.060; WS_CSU: *r* = −0.132). Linear regression plots (Fig. 2) also showed better fit for the ground seed models. Ground seeds showed stronger agreement with wet chemistry data based on preliminary comparisons and were used for the remaining accessions and analyses.

### Descriptive statistics

For the full set of 287 accessions, CP content estimated using NIRS calibrated with the GS LH equation ranged from 23.33% to 31.35%, with a mean of 27.31% and a median of 27.34% (Table 1). The SD was 1.48%, resulting in a CV of 5.41%. The distribution of CP was approximately normal (Supplementary Fig. 1), which supports the suitability of this trait for downstream genetic analysis and association mapping. The estimated broad-sense heritability (*H*^2^) for CP content based on LH NIRS values was 0.80.

**Table 1.** Descriptive statistics for crude protein content (%) in cowpea accessions based on NIRS calibration equations (legume hay vs cowpea-specific).

Equation	*n*	Mean	Median	SD	Min	Max	CV (%)
Wet chemistry	103	26.53	26.82	3.09	18.58	37.85	11.66
LH	287	27.31	27.34	1.48	23.33	31.35	5.41
Cowpea	287	25.85	25.8	2.53	18.66	34.88	9.77

LH, legume hay NIRS calibration; Cowpea, cowpea-specific NIRS calibration; SD, standard deviation; CV, coefficient of variation; *n*, number of accessions.

Wet chemistry data from 103 accessions showed wider variation in CP content. The mean protein content was 26.53%, with a median of 26.82% (Table 1). The values ranged from 18.58% to 37.85%, with SD of 3.09% and a CV of 11.66%. This wider variation does justify the selection of calibration samples ranging from low, medium, and high CP levels to improve the reliability of the NIRS prediction model.

### Development and validation of a cowpea-specific NIRS calibration model for CP prediction

**Fig. 3.**
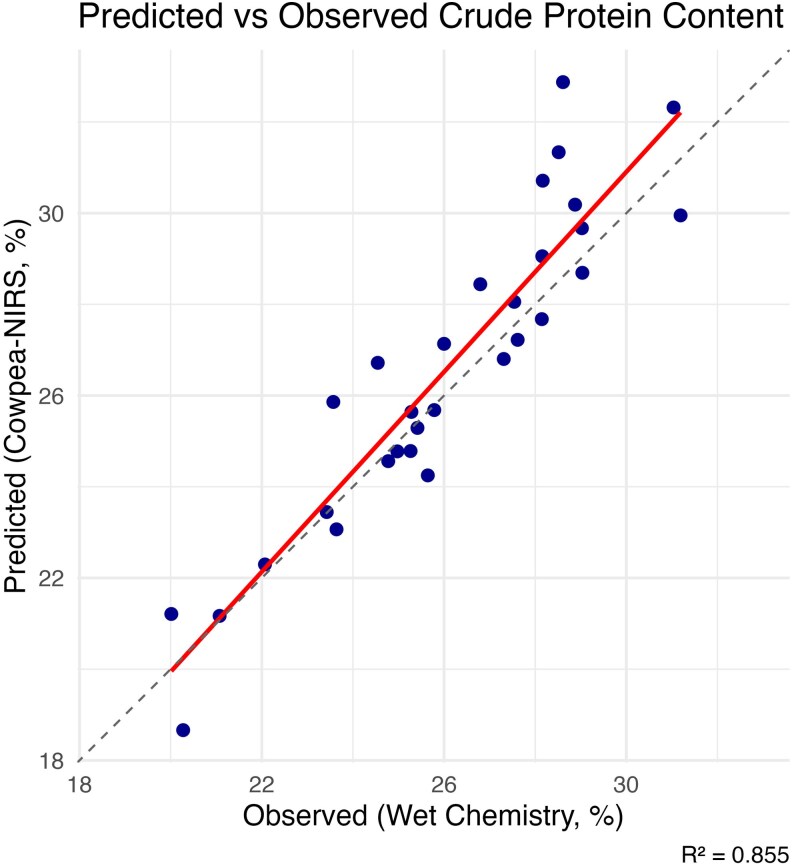
Performance of the cowpea-specific partial least squares regression (PLSR) calibration model for predicting crude protein content using NIRS data. The plot shows the relationship between reference crude protein values obtained by wet chemistry and values predicted by the cowpea-specific NIRS model for the validation set. Model performance metrics include Pearson's correlation coefficient (*r* = 0.92) and coefficient of determination (*R*^2^ = 0.86). The solid line represents the fitted regression line, while the dashed lined represents the 1:1 line (*y* = *x*).

The cowpea-specific NIRS calibration model showed a high predictive performance for CP content. In the validation set, the model had a correlation coefficient of 0.92 with a corresponding *R*^2^ value of 0.86 in the validation set (Fig. 3). We selected the model with 6 components as optimal based on the highest mean predictive correlation (*r* = 0.95) and lowest RMSEP (1.49), which balances model performance and parsimony while minimizing overfitting.

The CP content of the cowpea accessions predicted using the cowpea-specific NIRS ranged from 18.66% to 34.88%, with a mean of 25.85% and a median of 25.80% and a wider distribution (SD = 2.53%, CV of 9.77%) compared to the LH equation (Table 1).

**Fig. 4.**
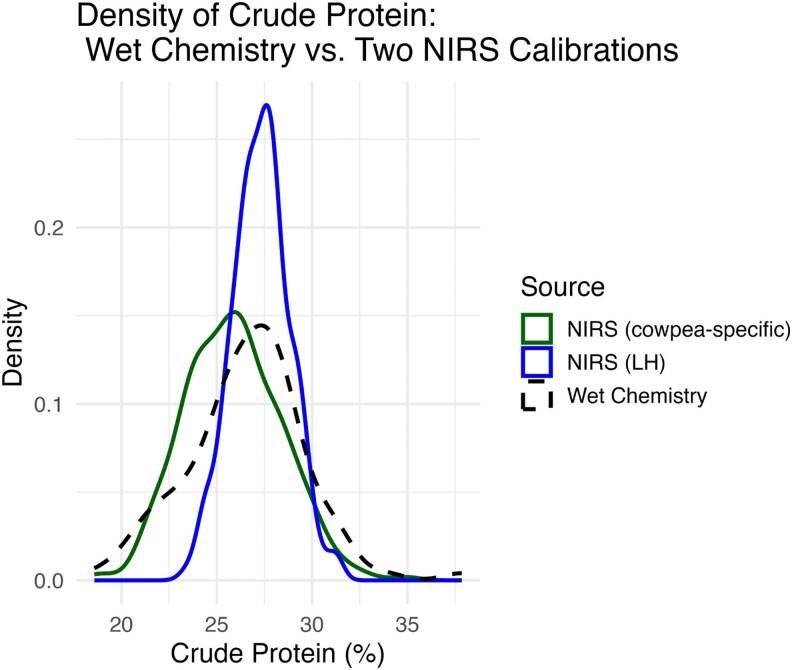
Density plot comparison of crude protein estimates derived from wet chemistry, cowpea-specific NIRS calibration, and legume-hay NIRS calibration across cowpea accessions. Curves represent the distribution of predicted crude protein values (%) for each method. The plot shows differences in the range and dispersion of protein estimates captured by the generic and cowpea-specific calibration models relative to wet chemistry reference values.

On average, our cowpea-specific model gave CP values that were 1.45% lower than those from the LH model. Despite this shift, the 2 methods showed a moderate-to-strong positive correlation (Pearson's *r* = 0.66, *P* < 2.2 × 10^−16^). A density plot comparison also showed key differences in distribution of the predicted values (Fig. 4).

The cowpea-specific model more closely aligned with the wet chemistry reference, as expected given that wet chemistry values were used for model development. This alignment was also shown in both the mean and dispersion of CP values, with the cowpea, with the cowpea-specific curve covering a wider phenotypic range than the LH calibration. In contrast, the legume-hay calibration had a narrower distribution and more central distribution. These visual patterns show the wider range captured by the cowpea-specific model, and an improved sensitivity to variation in CP content. Differences were also evident in the reference accessions used as checks which showed notable changes in magnitude between both calibrations, highlighted in the histograms (Supplementary Figs. 1 and 2).

Predictions from the plot-first and accession mean BLUE pipelines were strongly correlated (*r* = 0.90), with only slight differences in scale (RMSE ∼ 1.57). This showed that the use of accession-level BLUEs for calibration did not substantially affect the accuracy of prediction. A comparison of accession-level predictions from both methods is shown in Supplementary Fig. 3. External validation using the independent greenhouse dataset showed that the cowpea-specific calibration had a stronger correlation with wet chemistry measurement (*r* = 0.83), than the LH calibration (*r* = 0.77). However, the cowpea-specific predictions showed a systematic downward bias relative to wet chemistry values. Detailed comparisons are provided in Supplementary Figs. 4 and 5 and Supplementary Table 1.

### Comparative GWAS results from cowpea-specific and generic NIRS protein estimates

GWAS were carried out using CP estimates from both the GS_LH and cowpea-specific NIRS calibration equations. The choice of phenotyping method had an influence on the detection of significant SNP-trait associations.

**Fig. 5.**
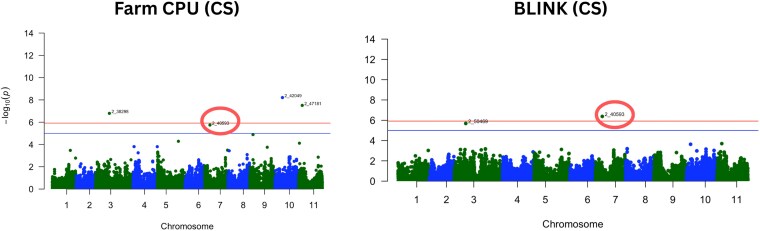
Manhattan plots of GWAS for crude protein content in cowpea using the cowpea-specific NIRS calibration under FarmCPU and BLINK models. The *x* axis shows physical positions across the 11 cowpea chromosomes, and the *y* axis shows −log_10_(*P*-values). The orange horizontal line indicates the genome-wide significance threshold based on Bonferroni correction (α = 0.05). SNP 2_40593, consistently detected on chromosome 7 across both models, is circled. The blue line indicates a suggestive threshold as implemented in the *qqman* package (−log_10_[1/number of markers]) and SNPs above the line were considered suggestive.

Using the cowpea-specific calibration with the FarmCPU model, 3 SNPs were statistically associated with CP (Fig. 5). We used Bonferroni correction for genome-wide significance and a suggestive threshold as commonly visualized in GWAS Manhattan plots (Turner 2018). The SNP *2_38298* on chromosome 3 (24,010,835 bp), which had a model-based PVE of 8.73%, SNP *2_42049* on chromosome 10 (13,068,722 bp), which accounted for 10.53% PVE and SNP *2_47181* on chromosome 11 (6,424,609 bp), contributing a PVE of 9.65%. An additional SNP, *2_40593* on chromosome 7 (9,133,335 bp), went past the suggestive threshold and was retained due to its consistent significance in both FarmCPU and BLINK models, explaining 7.36% of the variance. When using the BLINK model with the same cowpea-specific NIRS data, 2 more SNPs were detected. SNP *2_40593* appeared again, now with a PVE of 8.19%, and SNP *2_50469* on chromosome 3 (15,507,085 bp), with 7.28% PVE.

**Fig. 6.**
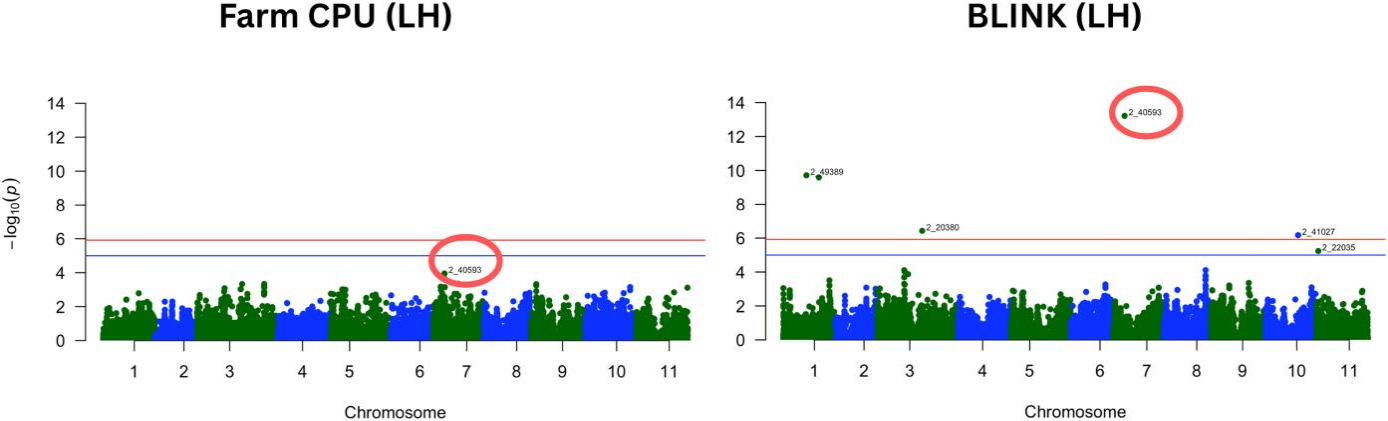
Manhattan plots of GWAS for crude protein content in cowpea using the legume hay (LH) NIRS calibration under FarmCPU and BLINK models. The *x* axis shows physical positions across the 11 cowpea chromosomes, and the *y* axis shows −log_10_(*P*-values). The orange horizontal line indicates the genome-wide significance threshold based on Bonferroni correction (α = 0.05). SNP 2_40593, consistently detected on chromosome 7 across NIRS sources, is circled. The blue line indicates a suggestive threshold as implemented in the *qqman* package (−log_10_[1/number of markers]) and SNPs above the line were considered suggestive.

In contrast, when using the LH model, no SNPs reached significance under the FarmCPU model. However, 5 SNPs were detected by the BLINK model (Fig. 6). These included SNPs *2_49389* on chromosome 1 (18,708,155 bp), with 12.38% PVE; SNP *2_43868* also on chromosome 1 (28,749,603 bp), contributing 12.23% PVE; SNP *2_20380* on chromosome 3 (36,377,388 bp), with a PVE of 8.25%; SNP *2_41027* on chromosome 10 (26,503,389 bp), with a 7.92% PVE; and *2_40593* on chromosome 7, which once again showed a showed a strong signal of 16.41% PVE (Table 2). Supplementary Fig. 1 shows quantile-quantile (QQ) plots for both GWAS models and calibrations. The observed values closely matched expectations under the null, which suggests effective control for population structure and supports the validity of the significant SNP associations.

**Table 2.** Summary of significant SNPs associated with crude protein content in cowpea, including chromosome locations (Chr), *P*-values, minor allele frequency (MAF), effect sizes, Watson-strand effect alleles (per Liang et al. 2024), and the model-based percentage of phenotypic variance explained (PVE%).

SNP	Chr	Position	*P*-value	MAF	Effect	Effect allele	Model-based PVE (%)	Model	Calibration
2_38298	3	24,010,835	1.61E-07	0.16898955	−0.7418	G	8.73	F	CS
2_42049	10	13,068,722	6.18E-09	0.1184669	−1.7153	G	10.53	F	CS
2_47181	11	6,424,609	3.11E-08	0.1010453	−1.6084	C	9.65	F	CS
2_40593	7	9,133,335	1.80E-06	0.12369338	−0.8381188	A	7.36	F	CS
2_40593	7	9,133,335	4.20E-07	0.12369338	−1.0535	A	8.19	B	CS
2_50469	3	15,507,085	2.07E-06	0.29442509	0.71724626	A	7.28	B	CS
2_49389	1	18,708,155	1.93E-10	0.05226481	−0.9311	G	12.38	B	LH
2_43868	1	28,749,603	2.56E-10	0.19163763	0.679	C	12.23	B	LH
2_20380	3	36,377,388	3.75E-07	0.1184669	−0.497	C	8.25	B	LH
2_40593	7	9,133,335	6.03E-14	0.12369338	−0.9484	A	16.41	B	LH
2_41027	10	26,503,389	6.77E-07	0.33275261	0.4632	G	7.92	B	LH

GWAS models (F = FarmCPU, B = Blink) and calibration methods used are also indicated.

Chr, chromosome; Position, physical position in base pairs; MAF, minor allele frequency; Effect, estimated allelic effect; Effect Allele, Watson-strand effect allele; PVE, phenotypic variance explained; F, FarmCPU; B, BLINK; CS, cowpea-specific NIRS calibration; LH, legume hay NIRS calibration.

Interestingly, SNP *2_40593* was statistically significant across both calibration methods and models (BLINK and FarmCPU). The MLMM model did not detect any significant associations under either calibration. The QQ plots of the GWAS models based on both sources are provided in Supplementary Figs. 6 and 7 with observations aligning with the expected null.

### Candidate genes

We identified candidate genes that are important for CP content in cowpea by examining the genomic regions around 20 kb significant SNPs using the IT97K-499-35 v1.2 genome assembly (Liang et al. 2024). The candidate genes showed expression in at least one tissue or developmental stage based on the transcriptome profiles from LIS (Dash et al. 2016). A subset of them showed valuable expression in developing seeds, nodules or vegetative tissues that are relevant to nutrient accumulation. Detailed list of gene names, positions, functional annotation and corresponding GO terms are provided in Supplementary Table 2.

For SNP 2_38298 (chromosome 3), 2 candidate genes *VuUCR779.03G183900* and *VuUCR779.03G184000* were found within the genomic region, both encoding a RING finger protein 38-like isoforms. SNP 2_47181 (chromosome 11) was linked to *VuUCR779.11G046700* and annotated as chitinase A-like protein. For SNP 2_40593 (chromosome 7), *VuUCR779.07G072100* was identified and corresponds to a cell division cycle protein 48 (CDC48) homolog.

Around SNP 2_20380 (chromosome 3), 3 candidate genes *VuUCR779.03G245800*, *VuUCR779.03G245900*, and *VuUCR779.03G246000* were identified, all containing zinc finger proteins or DNA-binding domains. At SNP 2_49389 (chromosome 1), *VuUCR779.01G079900* was identified, which codes for a hydrolase-related protein. At SNP 2_50469 (chromosome 3), multiple genes were identified, including *VuUCR779.03G151200*, which encodes a DNA-binding protein.

More regions include SNP 2_41027 (Chromosome 10), located near 2 genes, including one that codes for alpha-galactosidase 2 and SNP 2_43868 (Chromosome 1), which is linked to *VuUCR779.01G154000,* an ethylene-responsive transcription factor and *VuUCR779.01G154100* associated with ATP binding and protein serine/threonine kinase activity.

### GO annotations

Our search discovered 14 candidate genes across the key loci, with 13 genes assigned functional GO terms. These GO terms assigned to candidate genes were classified into molecular function and biological process categories. Common molecular function terms included protein binding (GO:0005515), zinc ion binding (GO:0008270), ATP binding (GO:0005524), and DNA binding (GO:0003677). Biological process annotations included carbohydrate metabolism (GO:0005975), DNA repair (GO:0006281), and chromosome organization (GO:0006310). The functional categories we identified, including transcriptional factors, ATP-binding proteins, carbohydrate metabolism enzymes and regulatory proteins are in line with previous literature in cowpea and other legumes where GWAS and QTL analyses have pointed to transcriptional regulators (eg NAC, WKRY, MYB, and TCP families) as well as metabolic enzymes that control seed development, nutrient transfer and storage compound biosynthesis (Lo et al. 2018; Huynh et al. 2024; Nkurunziza et al. 2025).

Several genes were associated with multiple GO terms. For example, SNP 2_40593, identified across both NIRS models on chromosome 7, corresponded to *VuUCR779.07G072100*, which had 5 GO annotations, including (GO:0005524) ATP binding, DNA repair (GO:0006281), and chromosome organization (GO:0006310) (Supplementary Table 2).

### REEU undergraduate training outcomes

All 4 REEU students successfully completed their assigned research training between 2022 and 2025. They contributed to generation and curation of datasets used in this study, including wet chemistry reference dataset of 103 accessions, 2 replicates. Also, in the preliminary NIRS benchmarking dataset of 56 accession, and the expanded full diversity panel of 287 accessions that then generated the standardized spectral datasets and phenotypic matrices used for cowpea-specific calibration model, descriptive statistics, and GWAS. Each cohort presented their project outcomes during the REEU final presentation symposium held at the end of the summer program. The 2025 Cohort was further involved in manuscript writing, adding to the integration of data interpretation and scientific communication.

## Discussion

### Value and reliability of NIRS for CP prediction in cowpea

NIRS continues to show itself as a powerful phenotyping tool in breeding programs. In this study, we further proved the application of NIRS for a faster and efficient estimation of CP across a diverse cowpea panel. Our comparative analysis between ground and whole seed scans confirmed that grinding the seeds significantly improves the accuracy of CP predictions, consistent with previous findings in legumes (Plans et al. 2011; Weng et al. 2017). Between the 2 commercial calibrations evaluated, the LH equation performed better than the CSU, but still underestimated CP content when compared to wet chemistry.

Our results support the claim that when NIRS is systematically applied, it can estimate CP with sufficient precision for follow-up applications like GWAS and GP. Our results also support earlier studies from other crops such as soybean (Yakubu et al. 2024), lentil (Hang 2022), and peanut (Govindarajan et al. 2009), where NIRS has proven useful for nutritional profiling. In addition, the speed and cost-effectiveness of NIRS make it especially suitable for breeding programs in low-income regions, where access to classical wet chemistry laboratories is limited (Hershberger et al. 2022; Abubakar et al. 2024). The relatively strong correlation (*r* > 0.80) between wet chemistry and ground-seed NIRS estimates in our study also validates its potential for early generation screening in cowpea. However, this performance depends on factors such as seed preparation, the quality of calibration equations, and sample heterogeneity (Font et al. 2006; Plans et al. 2011; Padhi et al. 2022), showing the importance of good protocols for improved accuracy in cowpea.

Our NIRS calibration equation (*R*^2^ = 0.86 and *r* = 0.92) aligns with previous research in cowpea, where models using PLSR have accurately predicted nitrogen content in seeds and leaves with *R*^2^ values in the 0.85 to 0.94 range (Towett et al. 2013; Muranaka et al. 2015; Padhi et al. 2022). For example, Biradar et al. (2024) reported *R*^2^ values up to 0.85 using whole seeds and ground seed PLSR calibration, Padhi et al. (2022)also reported values up to 0.93 in cowpea, showing the capability of NIRS capability for high-throughput screening in legume breeding programs. This confirms that ground-seed samples aligned more closely with wet chemistry estimates than whole-seeds, confirming that homogenized samples have the tendency to improve calibration accuracy. External validation using the independent greenhouse dataset further supported the practical utility of the cowpea-specific calibration. The model showed stronger agreement with wet chemistry values than the LH calibration, although predictions showed a consistent downward shift. Because accession-level summarization would have reduced this validation set to only 5 observations, model performance was assessed at sample level. Similar systematic deviations between calibration and independent dataset have been reported in NIRS studies are usually associated with differences in sample composition, calibration population structure or environmental conditions (Locher et al. 2005; Garcia et al. 2025).

### SNP-trait associations and candidate genes for protein content in cowpea

With the increasing availability of high-throughput SNP genotyping and trait-specific calibration tools, GWAS continues to be a powerful approach to look through the genetic basis of nutritional traits in legumes. Different studies have used GWAS to detect loci associated with important traits in cowpea, such as seed morphology (Lo et al. 2018), seed coat color (Lay et al. 2024), root traits (Zaffar et al. 2024), biological nitrogen fixation (Nkurunziza et al. 2025), particularly using the UCR Minicore (Muñoz-Amatriaín et al. 2021; Paudel et al. 2021; Andrade et al. 2023; Akinmade et al. 2025) showing its suitability for gene discovery. However, only a few have studied CP content and to our knowledge, this is the first GWAS using the UCR Minicore for this trait. Our analysis identified significant SNPs for CP content on chromosomes 1, 3, 7, 10, and 11, which indicates distributed genomic control across the crop's genome. Our findings can be linked with earlier QTL and GWAS studies in cowpea, which can point to potential shared genomic regions that influence nutritional and agronomic traits.

Candidate genes were identified by screening a ± 20 kb window surrounding each significant SNP using the cowpea reference genome (Liang et al. 2024). We selected this window size based on the observed pattern of LD from previous studies with the same accessions. Specifically, Andrade et al. (2023) reported that LD decayed to *r*^2^ = 0.15 across relatively compact intervals, supporting the use of narrow thresholds for GWAS follow-up. Our previous work (Akinmade et al. 2025) also used a narrow 10 kb window for seed and pod traits. In this study, we adopted a 20 kb window for better capture of candidate genes and without sacrificing resolution. Other studies, including Xiong et al. (2024), have used a similar method in searching for candidate genes associated with seed coat traits in cowpea. Candidate genes are presented as hypothesis-generating rather than confirmatory evidence. Functional annotations and GO terms were retrieved, and genes with expression patterns and biological functions that suggest roles in nitrogen accumulation, protein mobilization or seed development were further chosen for discussion.

On chromosome 3 we detected a significant SNP (2_38298, ∼24.0 Mb) and falls within a region previously associated with identified QTLs for pod shattering (*CPshat3*) and pod length (*CPodl3*) by Lo et al. (2018). Similarly, Sodo et al. (2025) reported minor QTLs for pod length (*qPodLt-3-1*) and peduncle length (*qPedLt-3-1*) in this region, suggesting that this region may contain genes with pleiotropic roles in both seed nutritional composition and plant architecture. Candidate gene analysis around this locus identified 2 genes: *VuUCR779.03G183900*, which encodes a basic helix-loop-helix (bHLH) DNA-binding protein, and *VuUCR779.03G184000*, a RING finger protein 38-like isoform. bHLH transcription factors are known to participate in nutrient signaling, mineral transport and seed development (Li et al. 2016; Guo et al. 2021; Zumajo-Cardona et al. 2023).

Another SNP on chromosome 3, 2_50469, was associated with *VuUCR779.03G151200*, a hAT dimerization domain-containing protein, annotated for DNA-binding activity (GO:0003677). Transposase-derived proteins like this one are sometimes adapted into functional roles within gene regulatory networks where they can have influence in the expression of genes during seed maturation (Feschotte and Pritham 2007).

On chromosome 7, SNP 2_40593 (9.13 Mb) was consistently identified across calibration methods and models. In Huynh et al. (2024), QTL *QSta.vu-7.1* was reported to be associated with stachyose content and subsequently mapped to the same region. Similar to that, Nkurunziza et al. (2025) detected significant marker effects on Chr7 (Vu07_34414059 and Vu07_34437020) associated with biological nitrogen fixation traits like nodule dry weight. These points to the possibility that the region around 9 to 10 Mb on Chr7 may control carbohydrate metabolism, nitrogen assimilation, and seed quality traits, which aligns with the central role of protein biosynthesis in these processes. Candidate gene analysis on SNP 2_40593 was repeatedly linked to *VuUCR779.07G072100*, a cell division cycle protein 48 (CDC48) homolog. This candidate gene had several GO annotations, including ATP binding (GO:0005524), DNA repair (GO:0006281), and chromosome organization (GO:0006310). Plant CDC48-like proteins have been reported to be involved in protein turnover, seed filling response to stress and metabolic homeostasis (He et al. 2020; Li et al. 2022; Lu et al. 2024), making this gene a good candidate for controlling nitrogen mobilization and seed protein composition.

On chromosome 10, SNP 2_42049 (13.07 Mb), identified using the cowpea-specific NIRS model and FarmCPU, had one of the highest effects, explaining 10.53% of the phenotypic variance. Another SNP on this chromosome, 2_41027 (26.50 Mb), was found using the LH model and explained 7.92% of the variance. While few studies have linked chromosome 10 to protein traits, Nkurunziza et al. (2025) reported markers related to nitrogen efficiency in this region, suggesting potential metabolic connections and calls for further exploration. Candidate gene analysis near SNP 2_41027 on chromosome 10 identified *VuUCR779.10G100600*, which encodes alpha-galactosidase 2, which functions in oligosaccharide hydrolysis at seed maturity (GO:0004553; GO:0005975). The alpha-galactosidase enzymes function in legume seeds to reducing antinutritional raffinose family oligosaccharides and balancing sugar-to-protein conversion (Obendorf and Górecki 2012; Arunraj et al. 2020).

On chromosome 11, our SNP 2_47181 (Chr11, ∼6.42 Mb) falls within the region where Huynh et al. (2024) identified QTLs affecting sugar-related traits such as sucrose, raffinose, and stachyose (*QSuc.vu-11.1* and *QSta.vu-11.1*). The overlap of sugar and protein related QTLs could point to the interconnected regulation of storage compound biosynthesis in cowpea seeds. The associated candidate gene *VuUCR779.11G046700* near this region encodes a chitinase A protein. Although typically associated with pathogen defense, chitinases also play roles in seed development and nutrient mobilization (Shobade et al. 2024).

On chromosome 1, we detected SNPs 2_49389 and 2_43868. Sodo et al. (2025) reported major QTLs for flowering time (*qNDFW-1-1*) and number of pods per plant (*qNpod-1-1*) on Chr1, while Huynh et al. (2024) also detected QTLs for calcium concentration and flowering time on this chromosome. These results signal to the notion of QTL hotspots where both protein accumulation and developmental traits may be controlled by shared regulatory genes. Candidate gene analysis near SNPs 2_49389 and 2_43868 on chromosome 1 identified transcriptional regulators including *VuUCR779.01G080000* and *VuUCR779.01G154000*, both of which are ethylene-responsive transcription factors, as well as *VuUCR779.01G154100*, which encodes a serine/threonine kinase. Ethylene signaling components and kinase cascades are known regulators of seed storage proteins synthesis and nutrient remobilization at seed development stages (Chen et al. 2023a, 2023b).

The MLMM was more conservative and did not detect these SNPs as significant, which is consistent with previous reports that show MLMM may underperform for traits controlled by multiple small-effect loci (Segura et al. 2012; Sandhu et al. 2024). Therefore, our use of FarmCPU and BLINK, which better accommodate complex trait architectures and reduce false positives without sacrificing power, remains a preferred option in nutrient trait GWAS (Liu et al. 2016; Huang et al. 2019).

The candidate genes lay a foundation for understanding the genetic control of protein content in cowpea and present candidates for functional validation and future marker development following validation. More than the candidate genes we identified in our GWAS, it makes sense to consider the biological processes like carbohydrate metabolism that act as a pathway of action for seed filling and protein accumulation. In the seed development phase, photosynthate transport and their allocation to protein, starch, and other reserves are mediated by enzymes within sugar metabolism and sucrose–starch conversions, regulating available carbon for biosynthesis of amino acid, and protein accumulation (Aguirre et al. 2018). If one of our significant SNPs is positioned close to the genes that code for sugar transporters or enzymes involved in glycolysis, they can influence the carbon supply for synthesis of seed protein, serving as a bridge connecting genotype to protein phenotype.

Adding to that, genome maintenance and post-translational regulation are important in the seed maturation phase and can have an effect on the final protein accumulated. Seeds experience damage to DNA (example strand breaks oxidative lesions) especially during storage and desiccation stages, their repair capabilities is crucial for growth and optimum metabolism reactivation during germination (Waterworth et al. 2022). Candidate genes that play a role in DNA repair pathways (such as homologous recombination) may have been overlooked but can be important in facilitating genomic integrity, which in turn influences overall seed viability and accuracy of metabolic activities. Also, protein phosphorylation is integral to regulation of seed metabolic activities. Studies have shown that many seed maturation proteins go through reversible phosphorylation, which affects enzymatic activity, signal transduction, and other metabolic activities associated with grain filling and maturation, as shown in soybean (Meyer et al. 2012). Therefore, SNP-linked candidate genes that code for kinases, phosphates, or phosphorylation proteins represent important functional candidates that may contribute to processes relevant to accumulation of protein in cowpea seeds.

### Relevance to breeding and biofortification

Our study provides new genetic information that can guide cowpea protein fortification and breeding protocols. The significant SNPs identified across 4 chromosomes show the important genomic regions that serve as starting points for downstream breeding work, including marker development and targeted validation. Our findings also add to the effort to include genomic tools into legume development programs that target nutritional traits (Hwang et al. 2014; Srungarapu et al. 2022). With additional confirmation in independent breeding materials, markers in these regions may be incorporated into selection pipelines to improve efficiency of developing biofortified cowpea varieties, especially in low-income regions that rely on plant-based protein for food and nutrition security. When these are further combined with farmer-prioritized traits, the cowpea varieties released will have a higher adoption potential (Boukar et al. 2019).

### Importance of protein improvement in cowpea forage systems

Cowpea forage is well known for its rich protein content, usually within a range of 23% to 32% (Abebe and Alemayehu 2022), making it a good alternative to soybean in poultry diets, especially in areas where cowpea is cheaper or more affordable than soybean (Bumhira and Madzimure 2023). Studies show that cowpea meal can substitute up to 20% of broiler feed without a compromise in its performance (Maidala and Dass 2017). Trials to compare feed also show cowpea protein digestibility (71% to 76%) is within the range of soybean meal (81% to 83%, and the addition of ∼10% cowpea grain can be advantageous (Bumhira and Madzimure 2023). In semiarid agro-pastoral farming systems, cowpea's dual-purpose as grain and good quality fodder has been optimized with breeding to support livestock feed and improve the overall farm productivity (Grings et al. 2010). Improving seed protein content can well better its feed value and reduce the need for supplementary protein sources in livestock feed rations, especially in places where soybean meal is less available.

### Usefulness of integrating undergraduate training through REEU

The addition of undergraduate research experience into genomics and phenotyping studies like this cowpea protein content analysis had several benefits. It promotes scientific research and discovery while building capacity of the next generation of scientists. The USDA-AFRI REEU program is designed to achieve this, by providing a platform for hands-on training in interdisciplinary agricultural research (USDA 2026). Such undergraduate involvement aligns with and further supports the evidence showing that early engagement in research increases student retention in STEM fields and helps to improve understanding of complex scientific concepts (Lopatto 2007; Shanahan et al. 2015). Our exposure of students to modern tools like NIRS and GWAS prepares them for careers that incorporate digital skills with agronomic solutions, a combination which is becoming increasingly critical in modern agriculture (Boz et al. 2024). The inclusion of students as coauthor also shows how mentorship and meaningful research involvement can have tangible scholarly outcomes.

## Conclusion

The results of our study show the combined utility of GWAS, phenomics, and student training to achieve nutritional trait improvement in cowpea. Through NIRS-based phenotyping with GWAS, we identified important SNP-trait associations and candidate genes underlying CP content across multiple chromosomes. Our development of a cowpea-specific NIRS calibration improved protein prediction accuracy, further validating the use of spectral tools for high-throughput breeding. Additionally, embedding this research within the USDA-AFRI REEU program shows the dual benefit of promoting crop science while equipping undergraduates with modern research skills. Our findings provide a strong foundation for future breeding efforts aimed at biofortified cowpea varieties through research results and capacity building.

## Supplementary Material

jkag088_Supplementary_Data

## Data Availability

The phenotypic data collected and used in this research are available in the Dryad Digital Repository under DOI: https://doi.org/10.5061/dryad.8cz8w9h72. The genotypic information is available in Muñoz-Amatriaín et al. (2021). Supplemental material available at G3 online.
