# Supplementary material for: Genetic dissection of protein content in cowpea using custom-made NIRS equations and GWAS as a model for nutritional breeding and undergraduate research training
Source: G3 (Bethesda). 2026 Apr 6;16(6):jkag088. doi: 10.1093/g3journal/jkag088 (PMC13232527; doi:10.1093/g3journal/jkag088)
Supplement: jkag088_Supplementary_Data [file jkag088_supplementary_data.zip › Supplemental_Material_List_G3-2026-406678.docx]

**List of supplementary data**

**Supplementary Figure 1.** Histogram of Crude Protein Estimates Using the Legume Hay (LH) Calibration Equation.

**Supplementary Figure 2.** Histogram of Crude Protein Estimates Using the Cowpea-Specific (CS) Calibration Equation.

**Supplementary Figure 3.** Comparison of accession-level crude protein predictions obtained from the plot-level cross-validation pipeline and wet chemistry BLUEs.

**Supplementary Figure 4.** Comparison of accession-level crude protein predictions obtained from the cowpea-specific NIRS calibration and wet chemistry measurements in the external validation dataset.

**Supplementary Figure 5.** Comparison of accession-level crude protein predictions obtained from the Legume Hay NIRS calibration and wet chemistry measurements in the external validation dataset.

**Supplementary Figure 6. QQ plots of GWAS for crude protein content using the cowpea-specific NIRS calibration under FarmCPU and BLINK models.** Observed vs. expected –log₁₀(p) values are shown to visually assess model fit and control for population structure.

**Supplementary Figure 7**. QQ plot of GWAS for crude protein content using the Legume Hay (LH) NIRS calibration under the BLINK model. Observed vs. expected –log₁₀(p) values are shown to visually assess model fit and control for population structure.

**Supplementary Table 1**. Performance metrics from external validation of crude protein predictions using the Legume Hay (LH) and cowpea-specific NIRS calibration models based on an independent greenhouse dataset.

**Supplementary Table 2. Candidate genes identified within ±20 kb of significant SNPs associated with crude protein content in cowpea.** Sheet 1 includes SNP positions, associated genes, expression data, genomic coordinates, GO terms, and enzyme classifications. Sheet 2 provides Gene Ontology (GO) molecular function annotations for the associated genes.
