# Supplementary figures and images for: Genetic dissection of protein content in cowpea using custom-made NIRS equations and GWAS as a model for nutritional breeding and undergraduate research training
Source: G3 (Bethesda). 2026 Apr 6;16(6):jkag088. doi: 10.1093/g3journal/jkag088 (PMC13232527; doi:10.1093/g3journal/jkag088)

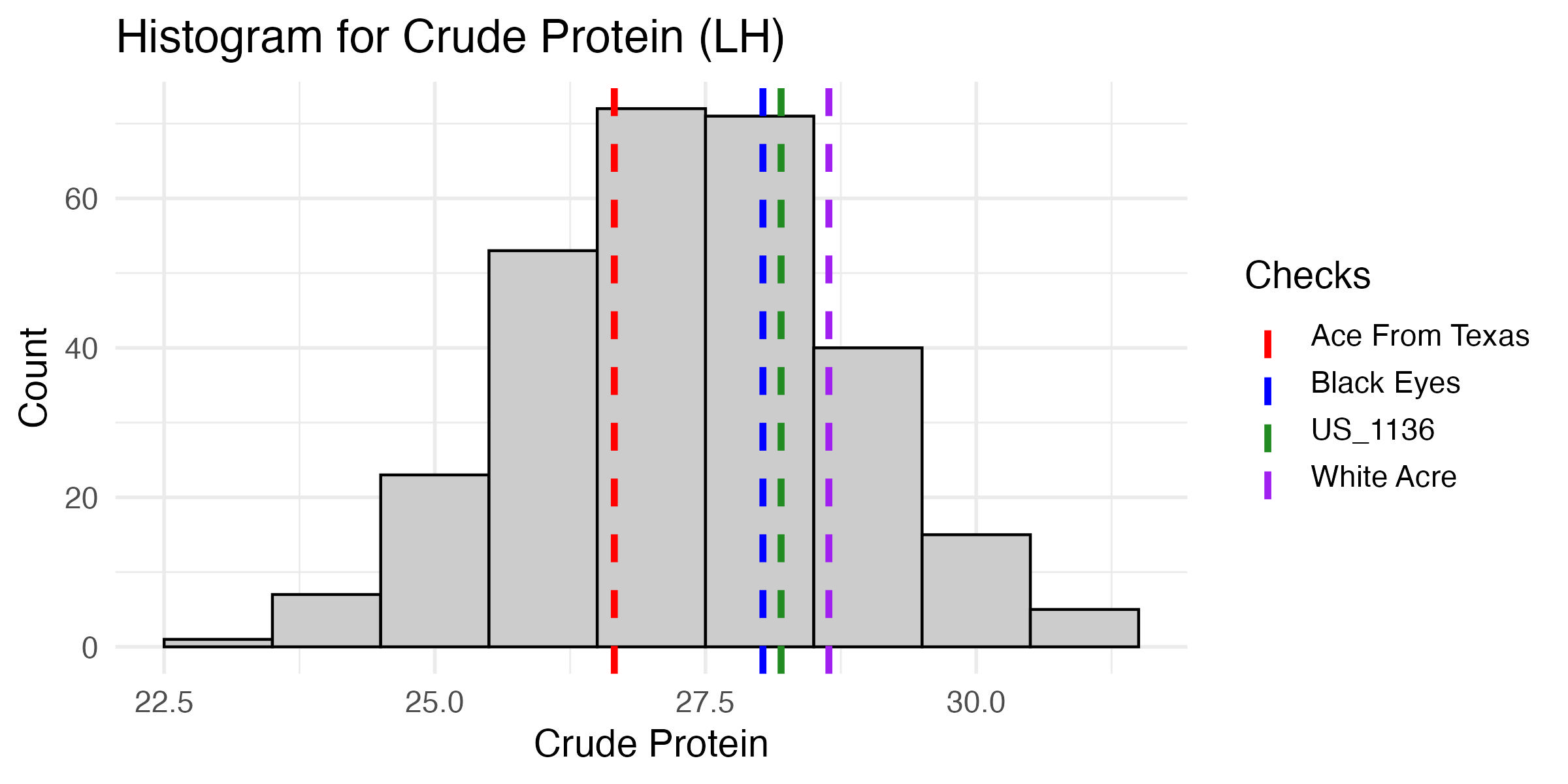

Supplement: jkag088_Supplementary_Data [file jkag088_supplementary_data.zip › Supplementary_Figure_1_G3-2026-406678.png]

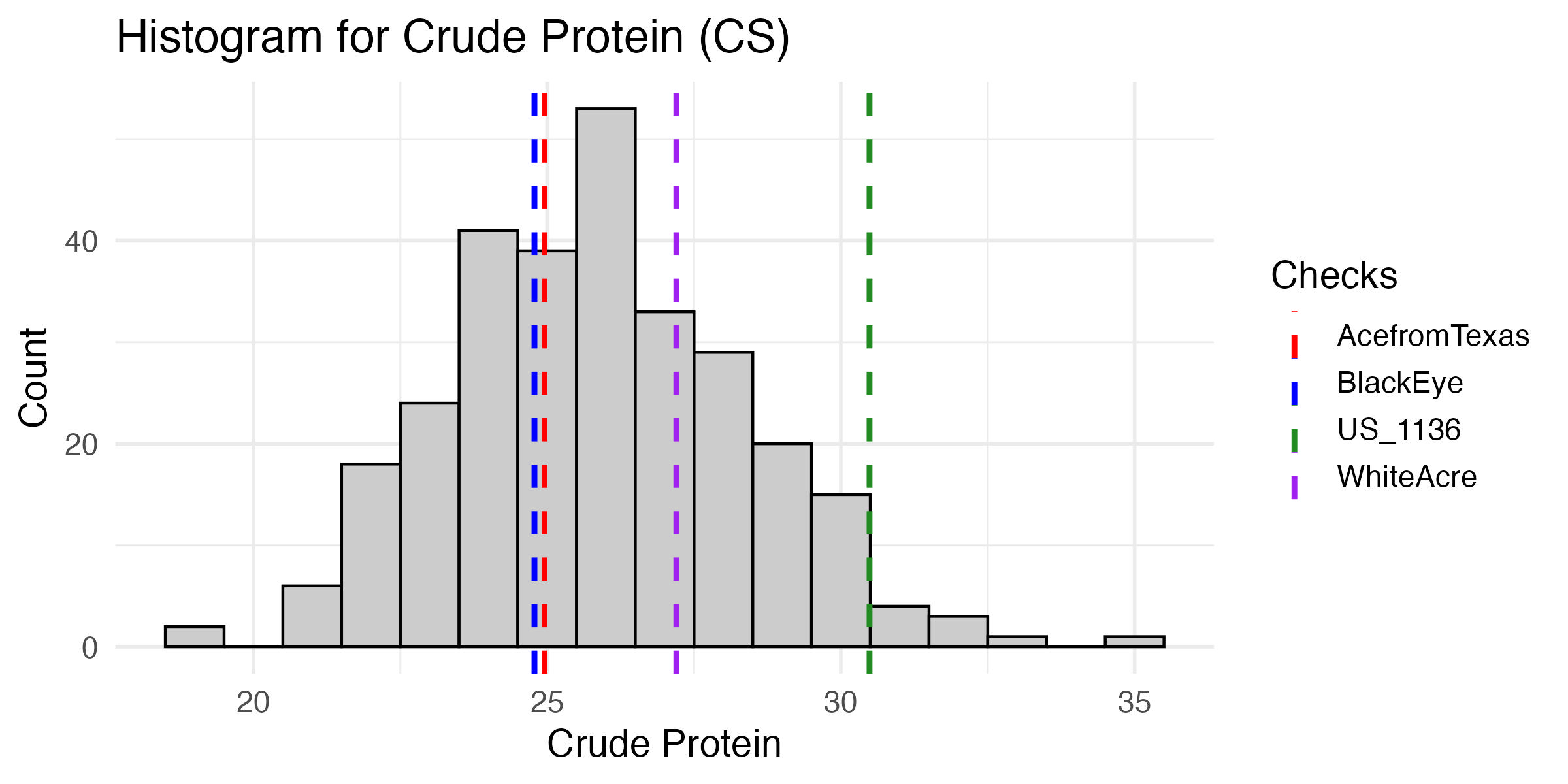

Supplement: jkag088_Supplementary_Data [file jkag088_supplementary_data.zip › Supplementary_Figure_2_G3-2026-406678.png]

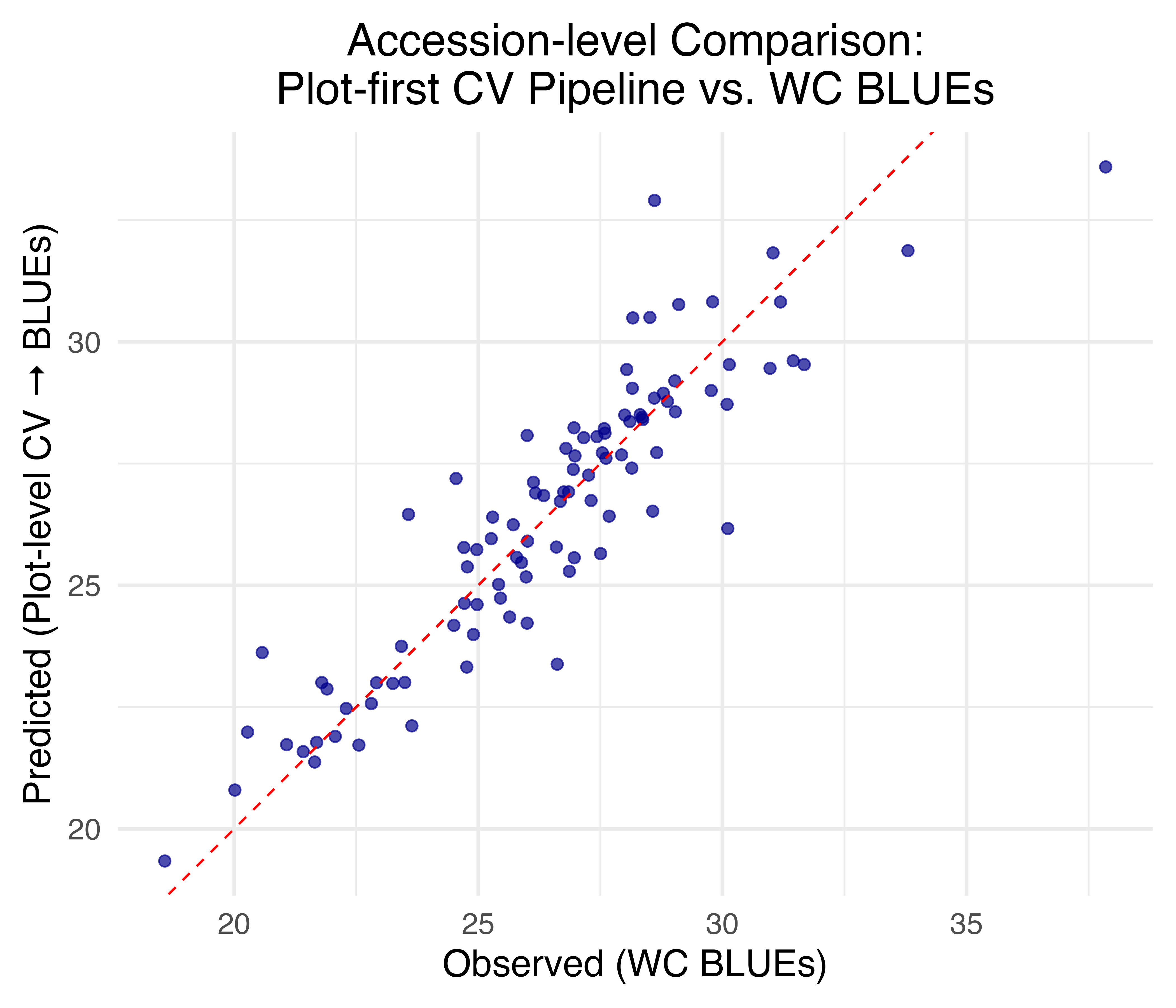

Supplement: jkag088_Supplementary_Data [file jkag088_supplementary_data.zip › Supplementary_Figure_3_G3-2026-406678.png]

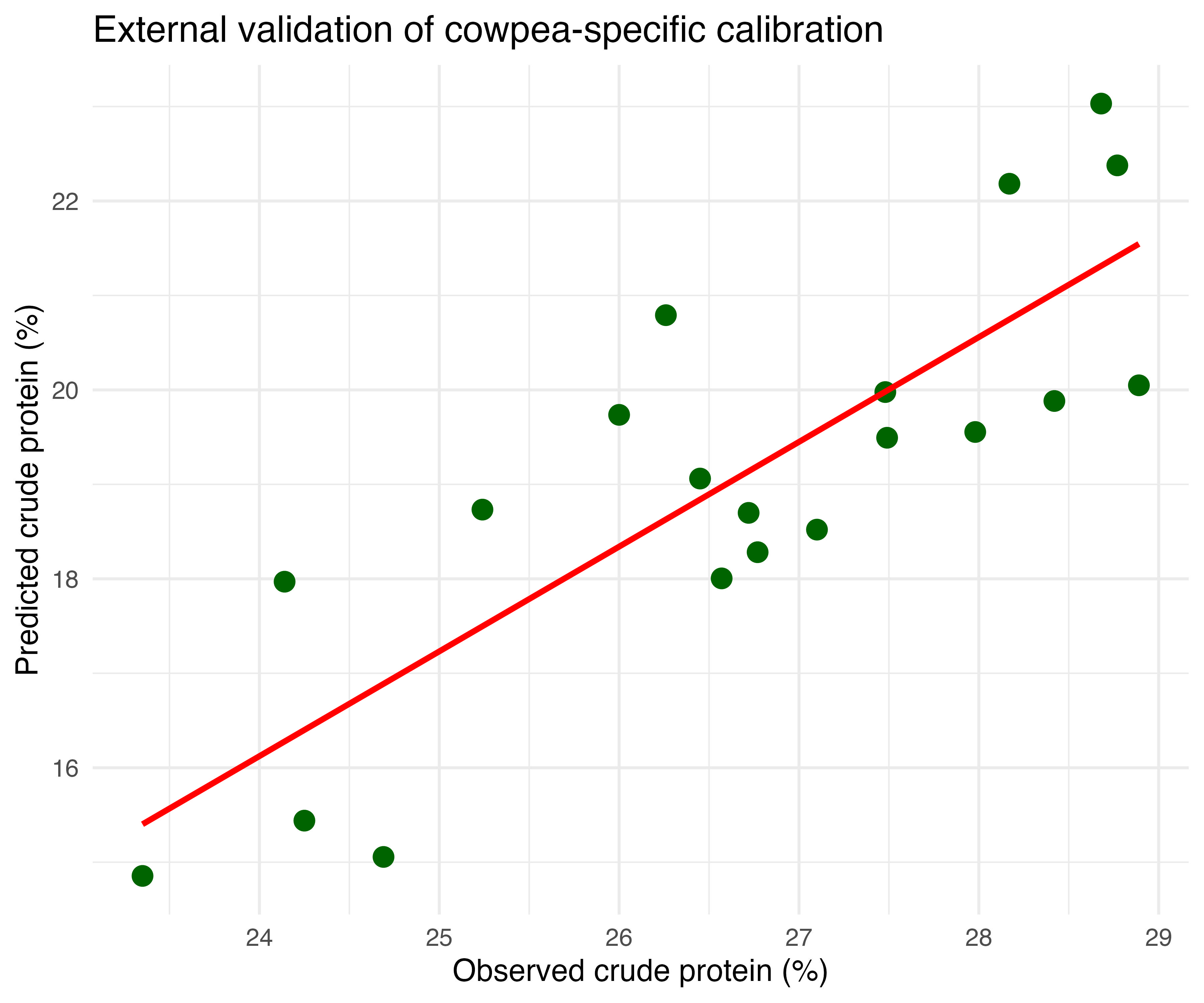

Supplement: jkag088_Supplementary_Data [file jkag088_supplementary_data.zip › Supplementary_Figure_4_G3-2026-406678.png]

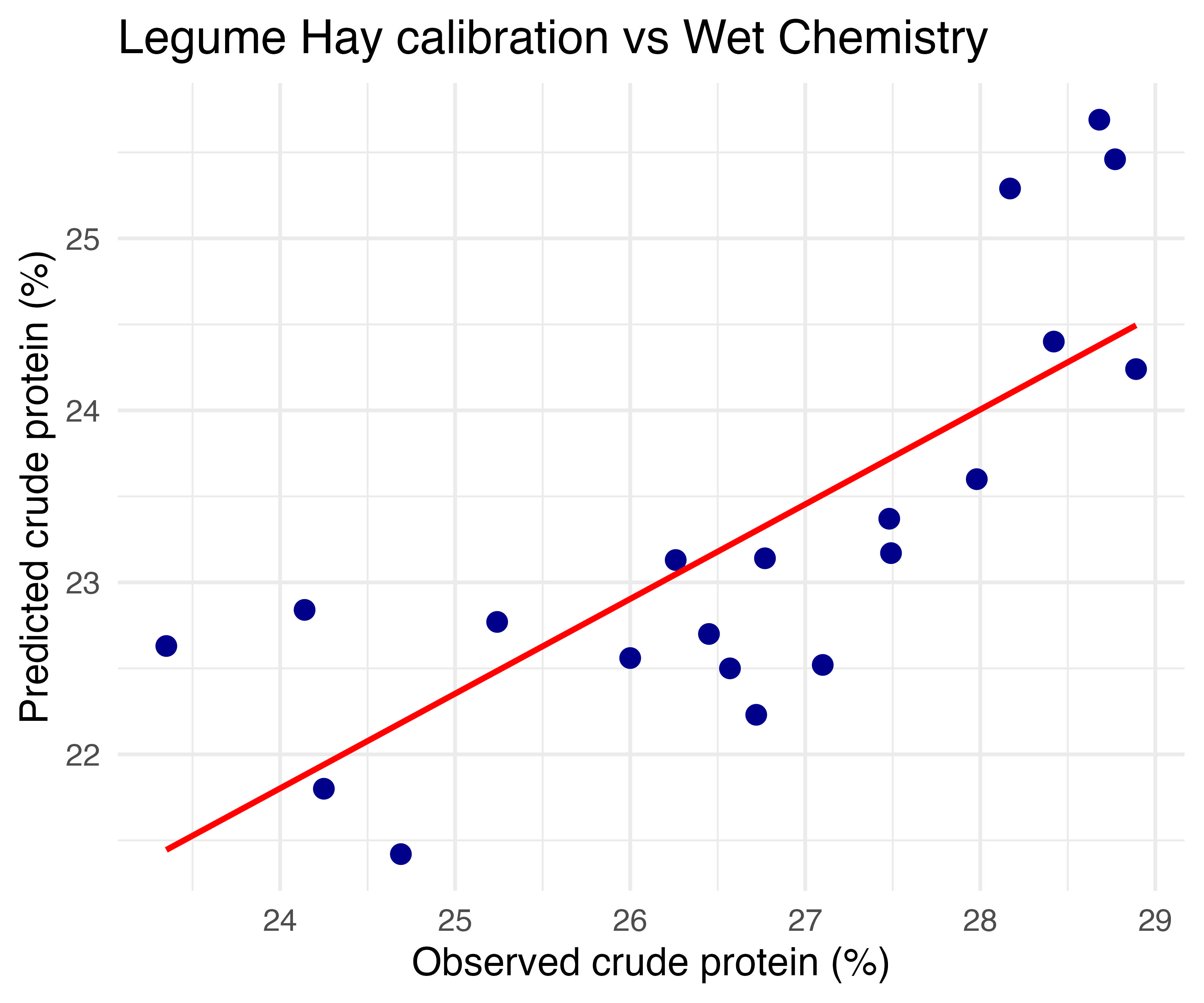

Supplement: jkag088_Supplementary_Data [file jkag088_supplementary_data.zip › Supplementary_Figure_5_G3-2026-406678.png]

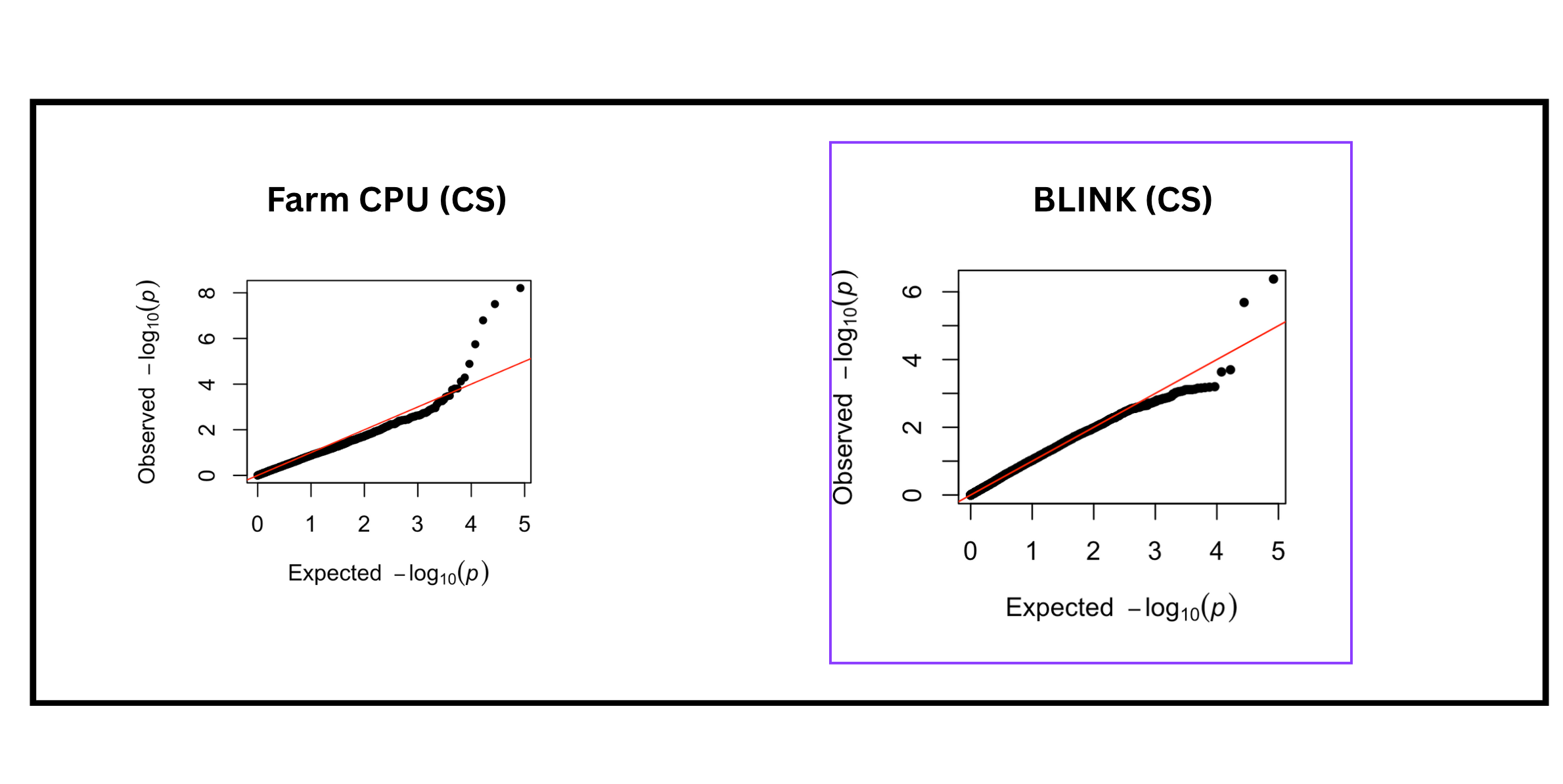

Supplement: jkag088_Supplementary_Data [file jkag088_supplementary_data.zip › Supplementary_Figure_6_G3-2026-406678.png]

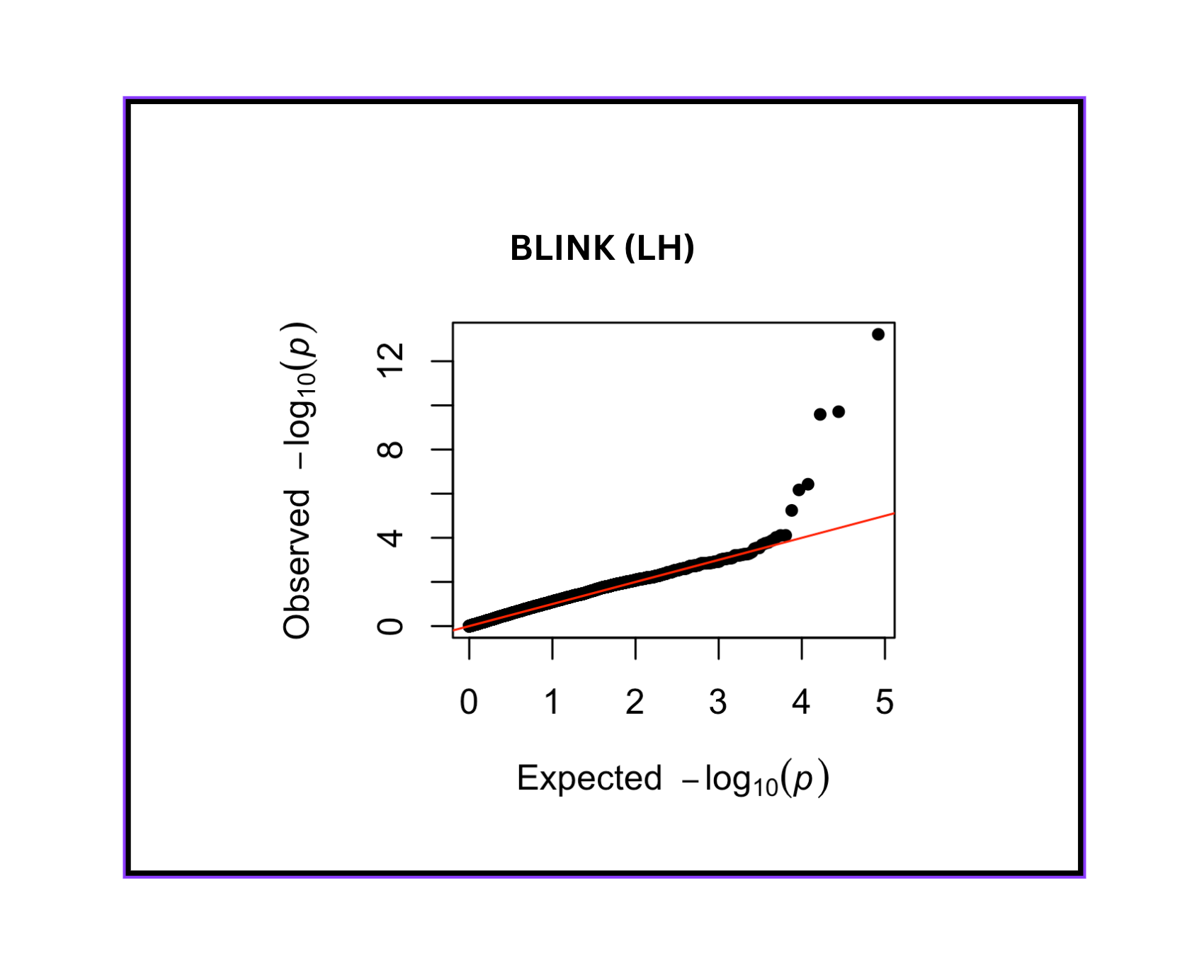

Supplement: jkag088_Supplementary_Data [file jkag088_supplementary_data.zip › Supplementary_Figure_7_G3-2026-406678.png]
